# Tabular Aids to the Recognition of Zoochemical Substances

**Published:** 1847-07

**Authors:** 


Art. IX.—*Hülfstabellen zur Erkennung zoochemischer Substanzen.* Von Dr. Freiherrn E. von Bibra.—*Erlangen,* 1846.

*Tabular Aids to the Recognition of Zoochemical Substances.* By Dr. F. E. von Bibra.—*Erlangen,* 1846.

Dr. v. Bibra has presented us, under this title, with four enormous sheets containing the reactions of all the important proximate constituents occurring in the animal body.

These tables give the chemical tests, physical characters, ultimate analyses, &c., of the following groups:

1. *Protein-compounds;* albumen, fibrin, casein, globulin, tritoxide of protein, binoxide of protein, and protein itself.
2. *Blood-pigments;* hæmatin, hæmaphæin, and hæmacyanin.
3. *Extractive matters;* those of flesh, blood, milk, and urine; osmazome, creatin.
4. *Gelatin;* glutin and chondrin.
5. *Horny matter;* keratin and chitin.
6. *Urine;* urea, uric acid, hippuric acid, uric oxide, cystin, kyestein, and allantoin.

7. *Urine-pigments ;* uroerythrin, cyanurin, melanurin, uroxanthin, uroglaucin, and urohodin.

8. *Bile ;* bilate of soda.

9. *Bile-pigment ;* biliverdin, biliphæin, and bilifulvin.

10. *Ptyalin, pyin, and pepsin.*

11. *Fats ;* stearin, margarin, olein, serolin, cholesterin, butyrin, castorin, cetin, aethal, hircin, and phocenin.

12. *Brain-fats ;* cerebrot, cephalot, cerebrol, and stearoconot.

13. *Sugar ;* diabetic sugar and milk-sugar.

14. *Organic acids ;* acetic acid, lactic acid, oxalic acid, lithofellinic acid, and bezoaric acid.

15. *Gases ;* oxygen, hydrogen, nitrogen, carbonic acid, carburetted hydrogen, sulphuretted hydrogen, and phosphoretted hydrogen.

16. *Sulphur and Phosphorus.*

17. *Inorganic acids ;* hydrochloric acid, sulphuric acid, nitric acid, hydrofluoric acid, carbonic acid, phosphoric acid, silicic acid, and hydrosulphocyanic acid.

18. *Bases ;* soda, potash, ammonia, lime, magnesia, alumina, iron, manganese, arsenic, and copper.

We extract his remarks on albumen as affording a fair illustration of his mode of treating his subject:

"*Albumen* (fluid). In all substances conveying fresh material for the nutrition of the organism, in blood, lymph, chyle, eggs, &c., also in morbid products. It coagulates in a tolerably concentrated solution in flocculi at 75° [169° F.], while in a dilute solution it gives rise to opalescence. Nitric acid, corrosive sublimate, and nitrate of the protoxide of mercury cause precipitates in extremely dilute solutions. Acetic acid does not precipitate it, but a precipitation occurs on the addition of ferrocyanide of potassium to a solution faintly acidulated by acetic acid. Alkalies prevent its coagulation by heat. Fluid albumen may be dried without assuming the coagulated state either *in vacuo*, or at a temperature not much exceeding 30° [86° F.] It is again soluble in water. Coagulated albumen: soluble in acetic acid, and again precipitable by exact neutralization with an alkali. It is thrown down from its acetic-acid solution on the addition of ferrocyanide of potassium. Coagulated albumen becomes blue if kept for some time in contact with concentrated hydrochloric acid."

Three ultimate analyses of albumen from different sources are appended. Suspended against a laboratory wall, these tables will undoubtedly be found useful by students of animal chemistry in the early part of their studies. Tables three feet long and two feet broad cannot be conveniently consulted in any other manner.

